# Porous Silk Fibroin/Cellulose Hydrogels for Bone Tissue Engineering via a Novel Combined Process Based on Sequential Regeneration and Porogen Leaching

**DOI:** 10.3390/molecules25215097

**Published:** 2020-11-03

**Authors:** Dennis Burger, Marco Beaumont, Thomas Rosenau, Yasushi Tamada

**Affiliations:** 1 Faculty of Textile Science and Technology, Shinshu University, Tokida 3-15-1, Ueda, Nagano 386-8567, Japan; 18hs110a@shinshu-u.ac.jp; 2 Institute of Chemistry of Renewable Resources, Department for Chemistry, University of Natural Resources and Life Sciences Vienna (BOKU), Konrad-Lorenz-Str. 24, 3430 Tulln, Austria

**Keywords:** silk fibroin, cellulose, biomaterials, bicontinuous composite, alkaline phosphatase, porous structure, MC3T3 differentiation

## Abstract

Scaffolds used for bone tissue engineering need to have a variety of features to accommodate bone cells. The scaffold should mimic natural bone, it should have appropriate mechanical strength, support cell differentiation to the osteogenic lineage, and offer adequate porosity to allow vascularization and bone in-growth. In this work, we aim at developing a new process to fabricate such materials by creating a porous composite material made of silk fibroin and cellulose as a suitable scaffold of bone tissue engineering. Silk fibroin and cellulose are both dissolved together in *N*,*N*-dimethylacetamide/LiCl and molded to a porous structure using NaCl powder. The hydrogels are prepared by a sequential regeneration process: cellulose is solidified by water vapor treatment, while the remaining silk fibroin in the hydrogel is insolubilized by methanol, which leads to a cellulose framework structure embedded in a silk fibroin matrix. Finally, the hydrogels are soaked in water to dissolve the NaCl for making a porous structure. The cellulose composition results in improving the mechanical properties for the hydrogels in comparison to the silk fibroin control material. The pore size and porosity are estimated at around 350 µm and 70%, respectively. The hydrogels support the differentiation of MC3T3 cells to osteoblasts and are expected to be a good scaffold for bone tissue engineering.

## 1. Introduction

Bone Tissue Engineering (BTE) investigates methods to regenerate bone for clinical application and tissue models. Grafts from BTE can be transplanted to alleviate and heal bone defects and thereby provide another option for current clinical treatments [1]. In general, the bone structure is comprised of dense cortical bone and, to some extent, sponge-like cancellous bone that contains pores filled with bone marrow or fat [1]. The bone structure consists of approximately 20% organic matter, 10% water, and 65% inorganic matrix [2]. The organic and inorganic matrix are mainly made up of an elaborate arrangement of collagen I fibers with hydroxyapatite [3]. Bone tissue is renewed and remodeled constantly by formation and resorption, which is performed by osteoblasts (bone forming) and osteoclasts (bone resorbing) [1,4]. For this reason, the recruitment of mesenchymal stem cells (MSCs), which are the progenitors of osteoblasts, their homing, and the ability of the surrounding tissue to elicit differentiation to the osteogenic lineage is of great importance for the repair of bone fractures [5]. Osteoblasts express alkaline phosphatase (ALP), which is an important enzyme involved in tissue matrix mineralization, and as they become encapsulated within their own matrix they are referred to as osteocytes [4,6,7]. These in turn produce substances that can modulate the recruitment, differentiation, and activity of osteoblasts and osteoclasts upon mechanical stimulation [1]. This makes osteocytes an essential mediator in directing bone remodeling in response to mechanical stimulation [8].

A BTE scaffold should favor cell differentiation to the osteoblastic lineage, which largely depends on the extra cellular matrix (ECM) as the regulator of stem cell fate [9,10,11]. Further tasks include guiding bone growth to desired areas and stimulating the integration of nascent bone tissue into the surrounding bone matrix [12]. To achieve this, the scaffold needs to fulfill several requirements, such as the appropriate mechanical strength that can sustain cell attachment and proliferation, good biocompatibility, biodegradability that matches tissue growth rate, and an interconnected porous structure that allows the flow of nutrients and waste to maintain cell viability [13]. To improve applicability, scaffold features such as molecular structure, embedding of cell signaling factors, surface treatment, mechanical properties, and micro/nanometer scale topography are varied to positively influence adhesion, migration, proliferation, differentiation, and cell signaling [10,14,15,16,17,18,19,20]. The choice of base material for the scaffold matrix that mimics mechanical and biological characteristics of natural bone matrix therefore presents the key challenge for BTE, which is why many materials have already been tested for BTE application [1,21,22]. Sponge-like scaffolds are favored, as the pores have been shown to support cell adhesion, proliferation, migration, and nutrient/waste flow. Pores are mostly induced by gas foaming, lyophilization, or porogen leaching, with their size being adjustable [23,24]. Various authors have reported on the optimum pore size for BTE with different conclusions, some ranging from 96 µm to 400 µm [25,26,27,28,29]. A minimum pore size of 300 µm has been established, though, as the minimum requirement for vascularization and bone ingrowth, which are imperative for success in tissue regeneration [28,29,30]. In addition to porosity, also stiffness is an important feature, which contributes to more than only the mechanical stability. MSCs have been confirmed to show a varying degree of cell adhesion and different differentiation lineages, depending on the elasticity of the substrate upon which they were cultured. A differentiation to neuronal lineage has been reported for soft matrices, whereas stiff matrices were shown to be osteogenic [10,31]. A nanoscale matrix surface has also been shown to govern stem cell fate, with disordered surfaces eliciting the expression of calcifying proteins, while cells cultured on smooth surfaces failed to do so [16].

Silk is a common fiber material that is composed of the two major proteins silk fibroin (SF) and sericin [32]. SF is a current subject of research as material for tissue engineering. The superior moldability of SF allows processing into foams, meshes, fibers and films, thereby enabling the material to suit a variety of target tissues and creating an environment for desirable cellular responses [33,34]. SF has very appropriate properties for tissue engineering regarding mechanical stability, biological interactions, biocompatibility, tunable degradability, and low inflammatory response, which is why it is used for targets, such as blood vessels, bones, tendons, ligaments, and cartilage tissue [34,35,36]. Another useful characteristic of SF is that it functions as a suppressor of the Notch pathway, which is involved in the down regulation of osteoblastogenesis, thereby clearly supporting bone growth [37]. A particular benefit of using SF for BTE is that its surface amide groups interact with MSCs and induce the expression of osteogenic mRNA [38]. Similarly the high β-sheet contents in SF have been shown to favor osteogenic differentiation of MSCs [39]. The β-sheet content varies with the processing method of SF [35,40] and a high content also coincides with an increase in degradation time in vivo [41].

In order to enhance the SF performance for BTE, several composite materials have been proposed and studied, among which, calcium phosphate-based inorganic components and collagen are components, due to their presence in vivo, and other materials, such as cellulose, have been employed to reinforce the scaffold [42,43,44]. Cellulose can be found in animals, bacteria, and plants. It is a renewable, biologically produced polymer with wide availability, which is why it is the basis of the pulp and paper industries, being used for a great variety of applications [45], such as textiles, paper, green plastics [46], or also drug delivery [47,48]. Cellulose has been researched for tissue engineering applications as well as wound dressings [49,50,51], and the in vivo biodegradability of regenerated cellulose has been confirmed [52].

The composition of SF and cellulose allows them to complement each other, as the mechanical strength of the scaffold materials has been shown to increase with increasing cellulose content [53,54,55], whereas cell viability and adhesion were improved upon using SF and cellulose together rather than cellulose alone [56]. SF/cellulose composites have been prepared in various manners based on organic and aqueous solvents as well as ionic liquids, with some of them having been investigated for their cell culture performance with promising results and success in in vivo application. However, these composites have some disadvantages as many were only developed for fiber or film application, had insufficient pore size, required regenerated SF as substrate, or needed high temperature treatment for dissolving SF [44,53,54,57,58,59,60].

The aim of this work is to develop a novel process to fabricate a composite of SF and cellulose with a porous structure suitable for BTE with improved mechanical properties through the use of cellulose. The composite preparation method used in this work is based on the *N*,*N*-dimethylacetamide/LiCl (DMAc/LiCl) solvent, NaCl as water-leachable porogen, and a novel sequential regeneration process by a combined water vapor and MeOH treatment.

## 2. Results and Discussion

### 2.1. Hydrogel Fabrication

**Figure 1:**
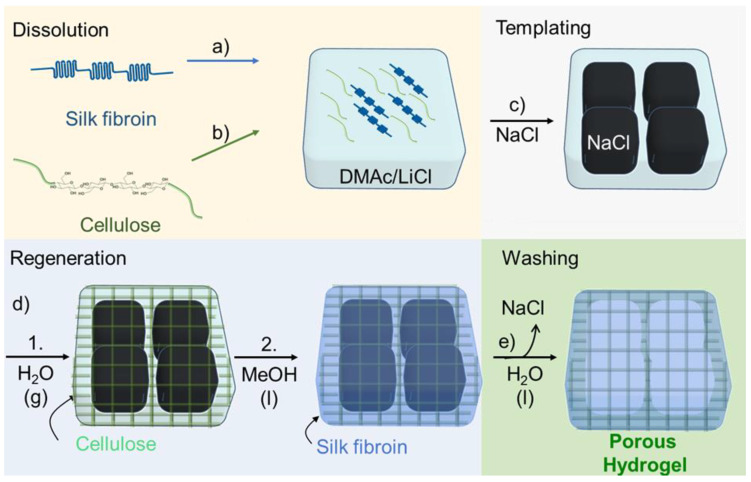
Hydrogel preparation. Dissolution: (a) Dissolving silk fibroin (SF) in DMAc/LiCl. (b) Dissolving cellulose in the SF solution. (c) Templating: Addition of NaCl powder. (d) Regeneration: 1. Cellulose is regenerated by water entering from air, gelation occurs; 2. soaking in methanol, SF is insolubilized. (e) Washing: Removal of porogen and solvent, a porous hydrogel is formed.

SF/cellulose hydrogels with porous structure were fabricated by employing a two-step, sequential gelation process. As shown schematically in Figure 1, SF was dissolved in DMAc/LiCl first and subsequently cellulose was dissolved in the SF solution (“Dissolution” in Figure 1). After adding NaCl powder as porogen (“Templating” in Figure 1), the solution was allowed to absorb minor amounts of water from the atmosphere of a defined humidity at room temperature. Acting as the antisolvent, the water caused gelation/regeneration of the cellulose, while SF was held in solution (“Regeneration Step 1” in Figure 1). At this stage, the binary solution turned to a gel structure around the porogen. This gel-like structure was immersed in methanol, which caused the insolubilization of SF through the crystallization of SF molecules (“Regeneration Step 2” in Figure 1), the solidification of the cellulose gel, and reprecipitation of residual dissolved cellulose. The final structure of the hydrogels was assumed as crystallized SF material embedded in the cellulose scaffold, both arranged around the NaCl porogen. These NaCl templates were removed through leaching in ROW (reverse osmosis water) at the final step (“Washing” in Figure 1). The samples were named S5C0.3, S5C0.5, and S5C1.0 according to the wt% contents of silk fibroin (S) and cellulose (C) in the “Dissolution” step. The control without cellulose was named S5C0.

### 2.2. Structural Analysis by FTIR

**Figure 2:**
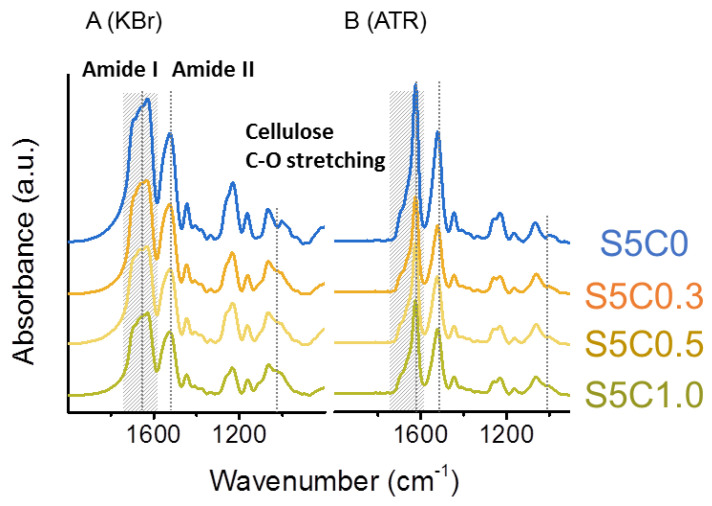
FTIR spectra of hydrogels. (**A**) Transmission (KBr) set up, (**B**) attenuated total reflectance (ATR) set up. Significant bands (amide I 1630 cm^−1^, amide II 1530 cm^−1^, cellulose C-O stretching 1019 cm^−1^) are marked by dotted lines. Amide I peak area for deconvolution is marked in hatching.

Figure 2A,B show FTIR spectra of SF/cellulose hydrogels measured in transmission mode with KBr pellets and attenuated total reflectance (ATR) mode, respectively. The amide I band at 1630 cm^−1^ and amide II band at 1530 cm^−1^, which are attributed to SF [55], were clearly observed in the spectra of SF/cellulose hydrogels and bands at 1019 cm^−1^ (C-O stretching band) and 1162 cm^−1^ (C-O-C stretching, glycosidic linkage) assigned to cellulose [61] also appeared in the spectra. The composition of both SF and cellulose was confirmed by this result. As all spectra were normalized to the cellulose-specific band area in the range of 1100 cm^−1^ to 960 cm^−1^, a decrease in the amide band area corresponding to an increase in cellulose content was observed.

Upon comparison of spectra taken in transmission mode (Figure 2A) and ATR mode (Figure 2B) a distinct difference in the peak shape of the amide I band can be noted. The amide I band reflects the secondary structure of proteins, such as SF. The amide I band of the spectra was deconvoluted to several peaks which are assigned to the secondary structures of SF. β-sheet, α helix/random coil, and turn/bend structures were assigned to peaks at 1622 cm^−1^ and 1639 cm^−1^, 1658 cm^−1^, and 1660~1700 cm^−1^, respectively [62]. The ratio of the secondary structures was estimated from the peak areas. Deconvolution of amide I bands is shown in more detail in Figure S2. Table 1 summarizes the results of both transmission mode and ATR mode measurements.

By transmission mode measurement, the β-sheet content for hydrogels was 32% to 37%. On the other hand, by ATR mode measurement, the cellulose-independent β-sheet content for hydrogels was estimated to be at around 64%. The ATR-reportable parts of the SF/cellulose hydrogels thus had a highly crystalline structure. The difference of β-sheet contents measured originates from the different penetration depths of IR transmission and ATR modes, which is why they react differently to the internal structure of the composites. The samples measured in both modes were powders prepared by crushing the freeze-dried hydrogels. While in transmission mode the IR light detects the structural information from the bulk composite, ATR mode provides the molecular structural information only from the surface-near region, because the IR light penetration depth is limited to several µm from the surface. We can estimate the penetration depth by Equation (1). [63]:

Penetration depth = λ/(2π√(sin^2^θ − (*n*_2_/*n*_1_)^2^)) (1)

we used 6.25 µm for λ at the amide I region wavelength (1600 cm^−1^). *n*_1_ and *n*_2_ are the refractive indices of diamond (2.376 at 6.25 µm) [64] and SF (1.530 at 1.5 µm) [65] used for calculation, respectively. θ is the incident angle of IR light to the prism, set at 45°. The resulting calculated penetration depth was 1.79 µm. Since the apparent powder size for ATR FTIR measurement was at several hundred µm, the ATR spectra were accumulated only from the near-surface region of the powder material.

The difference in β-sheet content obtained by ATR and transmission FTIR measurements can likely be attributed to the solvent penetration dynamics of methanol in the composite, which seem to favor surface-near regions. These results indicate that the SF/cellulose hydrogels fabricated by our process have a characteristic structure of non-uniform crystallinity.

Due to this sequential regeneration step, we assume further the presence of a cellulose framework structure embedded into a silk fibroin matrix. The regeneration with water vapor causes the formation of a mechanically robust cellulose scaffold, which is a typical observation for cellulose gels prepared by comparable processes [66,67]. We further confirmed this structure, by washing the hydrogel after this treatment directly with water without the additional MeOH step (thereby dissolved silk was removed), and the IR spectrum of this cellulose-rich sample is shown in Figure S3. In the process with the MeOH regeneration step, SF is immobilized, surrounding the cellulose-rich network. Due to this special bicontinuous SF/cellulose composite structure, we also expected differences in the cellulose/silk ratio determined from IR measurements at the sample surface (ATR mode) and the whole bulk sample (transmission mode), which are clearly shown in Figure S4.

### 2.3. Structural Characterization and Mechanical Properties of the Cellulose-SF Hydrogels

**Figure 3:**
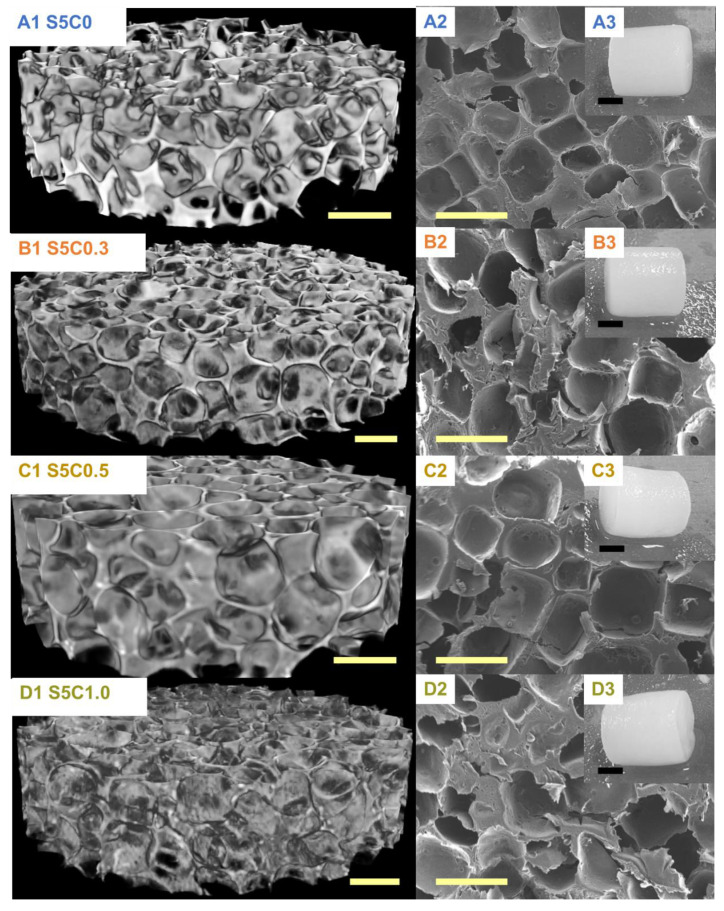
µCT images (**A1**–**D1**) and SEM photographs (**A2**–**D2**); yellow scale bar, 500 µm. Appearances of SF/cellulose hydrogels and control (**A3**–**D3**); black scale bar, 5 mm. **A**: S5C0, **B**: S5C0.3, **C**: S5C0.5, **D**: S5C1.0.

The hydrogels have a uniform porous structure with homogenous surface and pore distribution independent of cellulose composition (Figure 3). The determined average pore size and porosity are summarized in Table 2. The pore size and the porosity of the SF/cellulose hydrogels were 340 to 370 µm and 73% to 74%, respectively. There is no significant difference of pore structure, size, and porosity among samples. These results indicate that the amount of cellulose in the hydrogel did not affect the pore structure, size, and distribution, which were solely determined by the NaCl porogen. Previous reports emphasized the importance of a minimum pore size of 300 µm to enable adequate tissue in-growth in 3D scaffolds [28,29,30]. The pore sizes in our hydrogels fulfill this requirement.

**Figure 4:**
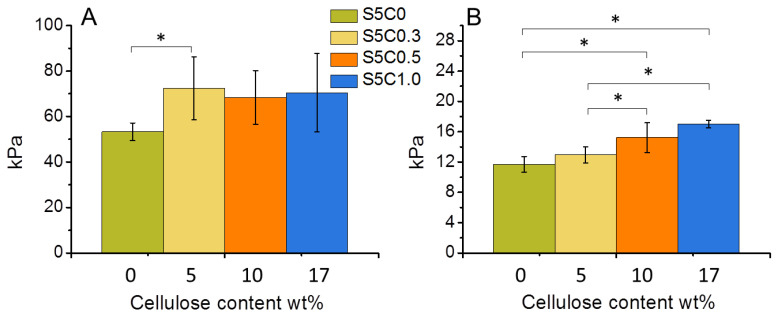
Change of compressive modulus (**A**) and compressive strength at 30% strain (**B**) of wet SF/cellulose hydrogels depending on the amount of cellulose. Significant difference (*p* < 0.05) is indicated by an asterisk symbol (*).

The compression properties of the hydrogels are summarized in Table 2, while Figure 4 shows the change of compression modulus and strength according to the amount of cellulose in the SF/cellulose hydrogels. The compressive modulus significantly increased by the first addition of cellulose to SF, but did not significantly increase further with higher cellulose contents. On the other hand, the compressive strength increased according to the amount of cellulose. The 17 wt% cellulose content showed an improvement of the compressive strength by a factor of 1.4. These results indicate that the presence of cellulose increases the mechanical strength and the stiffness of the SF material. Representative stress-strain curves measured for all samples are shown in Figure S1. The elastic modulus of cell substrates has been reported to be a directing factor to determine stem cell fate. As the material stiffness contributes to the osteogenic ability of BTE scaffolds [10,31], cellulose addition causes an important improvement of the osteogenic performance of the SF material.

### 2.4. Biological Properties of the Cellulose/SF Hydrogels

**Figure 5:**
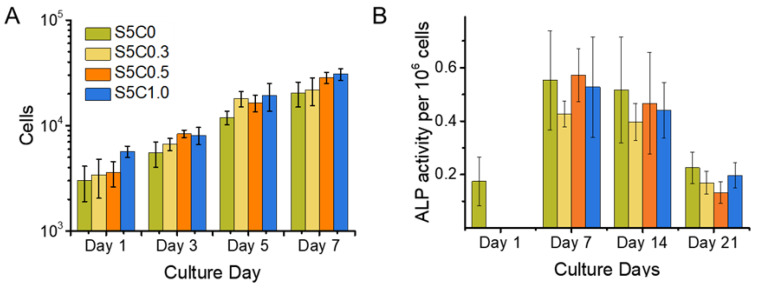
Cell Proliferation and alkaline phosphatase (ALP) Expression. (**A**) Cell proliferation of MC3T3 cells on hydrogel and control sample scaffolds during a time span of 7 days. Cell number is shown in logarithmic scale (**B**) ALP activity of MC3T3 cells cultured on hydrogel (S5C1, S5C0.5, S5C0.3) and control (S5C0) samples over a time span of 3 weeks. Cell proliferation was observed simultaneously with the same experimental set up in order to obtain cell numbers for normalization of measured ALP activity.

Initial cell adhesion and cell proliferation experiments were performed on the hydrogels and the SF control (S5C0) samples. As shown in Figure 5A, no significant differences among the samples with regard to initial cell adhesion were observed on day 1. SF has been reported to be a good substrate for supporting cell adhesion. The good cell adhesion of the SF control sample was maintained in the case of the hydrogels with cellulose, which had similarly good cell adhesion abilities.

Figure 5A also shows the proliferation curve of MC3T3-E1 cells cultured on the hydrogels and the SF control samples. The cell proliferation profiles on all samples were similar. Good cell growth on the hydrogels was observed, the same as for the SF sample. The doubling time for cell growth calculated at logarithmic growth was estimated to be around 2 days. This is in agreement with the reported typical time of around 1 to 2 days [68]. Thus, the hydrogels showed the same biocompatibility as SF.

The function of ALP in bone tissue is to catalyze the hydrolysis of extracellular pyrophosphates and to increase the local concentration of inorganic phosphates, which elicits biomineralization [69]. ALP expression is upregulated for osteoprogenitor cells and osteoblasts, and decreases with ongoing differentiation to osteocytes, and no ALP expression occurs in mature osteocytes where the matrix has already mineralized [69,70].

Figure 5B shows the ALP activity for a calculated amount of 1 × 10^6^ cells for all samples and the SF control (S5C0) over a time span of 3 weeks. Samples were taken on the 1st, 7th, 14th, and 21st day after seeding, while the ALP activity was measured and the cell number counted. There was no relevant difference between ALP activity between the samples. This result indicates that the presence of cellulose in the hydrogels did not impair the advantage of SF on MC3T3 cell differentiation to osteocytes. Figure 5B shows a maximum ALP activity around 7 days after seeding. From this result, it can be concluded that osteoblast differentiation to osteocytes was well progressing until at least 7 days of culture on the hydrogels, while other studies reported that a decrease of ALP expression was observed after 14 days of culture on cross-linked SF gelatin scaffolds [39] and 21 days of culture on water based, methanol treated SF scaffolds [71]. Evidently, an acceleration of MC3T3-E1 differentiation to osteocytes occurred on our SF/cellulose hydrogels.

We propose that the mechanism of the earlier differentiation of MC3T3-E1 cells is due to the presence of low molecular-weight SF which is retained in the hydrogels, as no dialysis process to make SF solution is used. Low-molecular weight SF has been reported to be an inhibitor of the Notch pathway, which is involved in the down regulation of osteogenesis and thereby also an up regulator of ALP expression at an earlier time. The study suggested that the specific length of polypeptides, a combination of particular amino acids or a particular mixture of peptides of low molecular-weight SF is osteogenic, whereas isolated single repetitive motifs of the heavy chain (GAGVGY, GAGAGY, GAGAGS) did not show the same effect as the peptide mixture [37].

## 3. Conclusions

SF/cellulose hydrogels with a defined porous structure were successfully developed by a new approach that uses a binary solution of predissolved, native, degummed SF and cellulose in DMAc/LiCl and NaCl powder as a porogen. The pore size and the porosity of the SF/cellulose hydrogels were 340 to 370 µm and 73% to 74%, respectively. They did not change with the cellulose contents in the hydrogels. The presence of cellulose improved the mechanical stiffness and strength of the SF material. The sequential regeneration favors the formation of a reinforcing cellulose framework, regenerated first by atmospheric humidity, upon which, in a second step, SF is assembled by the action of methanol as the antisolvent. This causes the formation of a reinforcing cellulose framework structure embedded in a SF matrix. In surface-near layers, the secondary structure of SF in the hydrogels assumed abundant β-sheet conformation, whereas deeper layers of the scaffold show decreased β-sheet contents. As in vivo SF degradation inversely scales with β-sheet content, this offers great potential for resorption of the material upon sufficient bone regeneration by the body. The cellulose framework/SF matrix structure maximized material dependent improvement of hydrogel properties, as the cellulose framework mainly contributed to mechanical property enhancement, while the SF matrix supported cell growth. Osteocyte differentiation of MC3T3-E1 cells cultured on the hydrogels was observed. Even more, the good differentiation properties of SF materials were not impaired by the incorporation of cellulose, and the osteocyte differentiation was accelerated compared to other SF substrates. These results show SF/cellulose hydrogels to be a suitable scaffold for BTE.

## 4. Materials and Methods

Dried bleached beech sulfite dissolving pulp (M_w_ = 303.7 kg/mol^−1^) was provided by Lenzing AG (Lenzing, Austria). Silk cocoons (Bombyx mori) were obtained from an experimental farm in Faculty of Textile Science and Technology, Shinshu University, Ueda, Japan. The used chemicals were obtained from Wako, Japan unless otherwise stated.

### 4.1. SF/Cellulose Hydrogel Preparation

#### 4.1.1. Dissolution

Silk (Bombyx mori) cocoons were cut into small pieces and degummed in 0.25 g/L Na_2_CO_3_ solution at 98 °C for 30 min. After washing with reverse osmosis water (ROW) the silk fibroin fibers were dried at 50 °C for storage. The fibers were washed consecutively with ROW, ethanol, dimethylsulfoxide (DMSO), and *N*,*N*-dimethylacetamide (DMAc) and then stored in DMAc for more than 12 h. The DMAc was removed by filtration under reduced pressure and the fibers were added to DMAc/LiCl (9 wt%). The suspension was kept stirring for 24 h at room temperature to yield a solution with a silk fibroin concentration of 5 wt%. In order to remove undissolved impurities, the solution was centrifuged for 5 min at 30,000× *g* rcf and the supernatant was further used. Dried cellulose pulp was swelled in water and blended by a mixer (Iwatani, IFM-800DG, 20,000× *g* rpm) at room temperature. The cellulose fibers were washed with ROW, ethanol, and DMAc and kept in DMAc for more than 12 h. After vacuum-filtration, the cellulose fibers were added to the 5 wt% silk fibroin solution in DMAc/LiCl, to yield cellulose concentrations of 0.3 wt%, 0.5 wt% and 1 wt% (*w*/*v*). Samples made with these solutions were named S5C0.3, S5C0.5, and S5C1.0, respectively. The solution was kept stirring for 24 h at room temperature.

#### 4.1.2. Hydrogel Preparation

Molds were prepared by cutting off the outer stamp and head of 10 mL disposal syringes (TERUMO Co. Ltd., Tokyo, Japan). The syringe body was filled with 3.6 g of NaCl powder (mean grain size: 440 µm, Wako Co. Ltd., Tokyo, Japan). Approximately 1 mL of the SF/cellulose solution was added to cover the NaCl in the syringe and the syringe was centrifuged at 30,000× *g* rcf for 5 min to settle the NaCl powder. The syringes were placed into an airtight vessel containing 100 mL of saturated MgCl2 solution to maintain a relative humidity of 33% [72]. The syringes were kept at room temperature for 7 days. The formed bodies were pushed out from the syringes and immersed in methanol for 24 h with shaking. The resulting composite bodies were soaked in ROW for at least 8 h and solvent exchange was repeated 10 times to completely remove remaining solvents (methanol and DMAc). During this process, the NaCl in the hydrogels was also dissolved and leached out. The hydrogels were kept in ROW and autoclaved for storage to prevent microbial growth. As control, a solution of 5 wt% SF in DMAc/9% LiCl without cellulose was prepared and processed as above except for solidification in gaseous methanol and not by aqueous humidity. The control sample was named S5C0.

### 4.2. Cell Proliferation and Adhesion

For the evaluation of cell adhesion and proliferation behavior on the hydrogels, MC3T3-E1 cells (RIKEN BRC, Tsukuba, Japan) which can differentiate to osteoblast-like cells, were cultured on the hydrogels. The cylindrical hydrogels were cut into disks with a thickness of 1–2 mm and disks with a diameter of 10 mm were punched out. These disks were autoclaved and placed into the wells of a 48 well cell culture plate. The suspended MC3T3 cells (5 × 10^4^ cells/100 µL in culture medium, Eagle MEM with 10% Fetal Bovine Serum) were seeded per well and incubated at 37 °C, 5% CO_2_. After 1 day of culturing, 900 µL of medium were added to each well. The hydrogels were taken on the 1st, 3rd, 5th, and 7th day after seeding, washed by PBS (−) buffer and immersed in 500 µL of 0.5% Triton X-100/PBS to make a cell lysate for cell counting by LDH activity assay. The LDH activity assay was performed according to a previous report [73].

### 4.3. ALP Expression Assay

For the evaluation of ALP expression of cells on the hydrogels, MC3T3-E1 cells were cultured on the disk hydrogels in the same manner as above described for the cell proliferation test. The culture was continued for 3 weeks and the hydrogels were taken on the 1st, 7th, 14th, and 21st day after seeding. As the samples were analyzed, cell number and ALP activity on the hydrogels were measured on the same day. TRACP & ALP assay kit (MK301, TakaraBio Co. Ltd., Tokyo, Japan) was used for determination of the ALP activity and the assay was performed according to the manufacture’s manual. Calf ALP (Lot No. 3628209, Toyobo Co. Ltd., Osaka, Japan) was used as a standard of ALP activity.

### 4.4. Pore Structure and Porosity

The hydrogels were lyophilized after solvent exchange with ethanol, followed by transfer to *tert*-BuOH. Dried samples were coated with platinum by vacuum evaporation (MSP-20UM, Vacuum Device, Mito-shi, Ibaraki, Japan) and observed by Scanning Electron Micrography (SEM) (Keyence VE-9800 SEM, Keyence, Osaka, Japan) at 10 kV. The average pore size was estimated by SEM photographs using ImageJ (v1.53a NIH, Bethesda, MD, USA).

The porosity of the hydrogels was estimated by µCT measurement. The measurement was performed by an X-ray Micro-tomograph apparatus (Skyscan-1272, Bruker, Billerica, MA, USA) at 5 µm/pixel resolution. Data sets were reconstructed (NRecon v1.7.4.6, Bruker, USA) and representative segments were transformed into binary images with dynamic thresholds and applied for morphometric analysis (CT Analyzer v1.18.8.0+, Bruker) and for making 3-dimensional models (CTvox v3.3.0, Bruker). The porosity was calculated during the morphometric analysis process by CT Analyzer.

### 4.5. Compression Test

The compression test was performed on a Tensile Test device (EZ-SX, EZ Test, Shimadzu co. Ltd., Tokyo, Japan). The cylindrical hydrogels (diameter 13 mm) were cut to a height of around 9 mm and kept wet during the compression test. The round compression area was 133 mm^2^ and the speed of compression was 5 mm/min. The compressive modulus was calculated from the initial slope of the stress-strain curve at 5% to 10% strain by Origin software (v9.65, OriginLab, Northampton, MA, USA). The compressive strength was determined from the stress at 30% strain, according to the literature [74,75].

### 4.6. FTIR

FTIR measurements were performed within the attenuated total reflectance (ATR) mode and transmission mode with KBr pellets (IRPrestige-21, Shimadzu Co. Ltd., Tokyo, Japan). The hydrogels were lyophilized with water in order to prepare them for measurement. The measurement ranges were 4000–600 cm^−1^, with an accumulation of 30 scans and resolution of 2 cm^−1^ for both modes.

The interpretation of the SF secondary structure was based on the analysis of the amide I region [62]. Briefly, a baseline was drawn between 1720 cm^−1^ and 1580 cm^−1^. By calculating the second derivative of the spectral data, information on bands attributable to specific secondary protein confirmations was obtained; Gauss-shaped curves were assumed for band integration. Band area ratios acquired through band deconvolution were used to calculate the relative structural content of secondary protein conformations. The peak analysis was performed by Origin software.

## Figures and Tables

**Table 1:** Secondary structure ratio of silk fibroin (SF) in the SF/cellulose hydrogel hydrogels by analysis of FTIR.

Content	Mode	S5C1	S5C0.5	S5C0.3	S5C0
β sheet, %	ATR	64 ± 3	65 ± 2	65 ± 2	65 ± 1
	Transmission	36 ± 1	36 ± 1	32 ± 7	37 ± 6
α-helix and random coil, %	ATR	18 ± 4	17 ± 2	19 ± 4	18 ± 2
	Transmission	35 ± 2	24 ± 4	29 ± 10	27 ± 12
Turns and Bends, %	ATR	17 ± 2	18 ± 2	17 ± 2	17 ± 1
	Transmission	29 ± 2	40 ± 5	39 ± 8	36 ± 7

**Table 2:** Porosity, pore size, and compression properties of hydrogels.

Property	S5C1.0	S5C0.5	S5C0.3	S5C0
Cellulose content [a], wt%	17	10	5	0
Porosity, %	74	71	73	74
Pore Size, µm	346 ± 106	339 ± 124	367 ± 104	345 ± 103
Modulus *, kPa	70.5 ± 17.2	68.4 ± 11.8	72.4 ± 13.9	53.3 ± 3.8
Compressive Strength *, kPa	17.0 ± 0.5	15.2 ± 2.0	12.9 ± 1.1	11.7 ± 1.0

[a] Estimated cellulose amount calculated from the respective cellulose and silk content in the precursor solutions. * Samples were measured in wet hydrogel state.

## Supplementary Materials

The following are available online, Figure S1: Representative stress-strain curves of all samples and control, Figure S2: Representative diagrams of deconvoluted peaks in amide I band region, Figure S3: Cellulose framework and SF matrix, Figure S4: Different bulk and surface composition.
