# Supplementary material for: Porous Silk Fibroin/Cellulose Hydrogels for Bone Tissue Engineering via a Novel Combined Process Based on Sequential Regeneration and Porogen Leaching
Source: Molecules. 2020 Nov 3;25(21):5097. doi: 10.3390/molecules25215097 (PMC7663655; doi:10.3390/molecules25215097)
Supplement: Supplementary file 1 [file molecules-25-05097-s001.pdf]

Supplementary Information

Porous Silk Fibroin/Cellulose Hydrogels for Bone Tissue Engineering via a Novel Combined Process Based on Sequential Regeneration and Porogen Leaching

Burger Dennis[1], Beaumont Marco*[2], Rosenau Thomas*[2], Tamada Yasushi*[1],

[1] Shinshu University, Faculty of Textile Science and Technology, Tokida 3-15-1, Ueda, Nagano 386-8567, Japan

[2] University of Natural Resources and Life Sciences Vienna (BOKU), Department for Chemistry, Institute of Chemistry of Renewable Resources, Konrad-Lorenz-Str. 24, Tulln, Austria

* Correspondence: ytamada@shinshu-u.ac.jp (Y.T.); thomas.rosenau@boku.ac.at (T.R.); marcobeaumont1@gmail.com (M.B.)

Compression test data

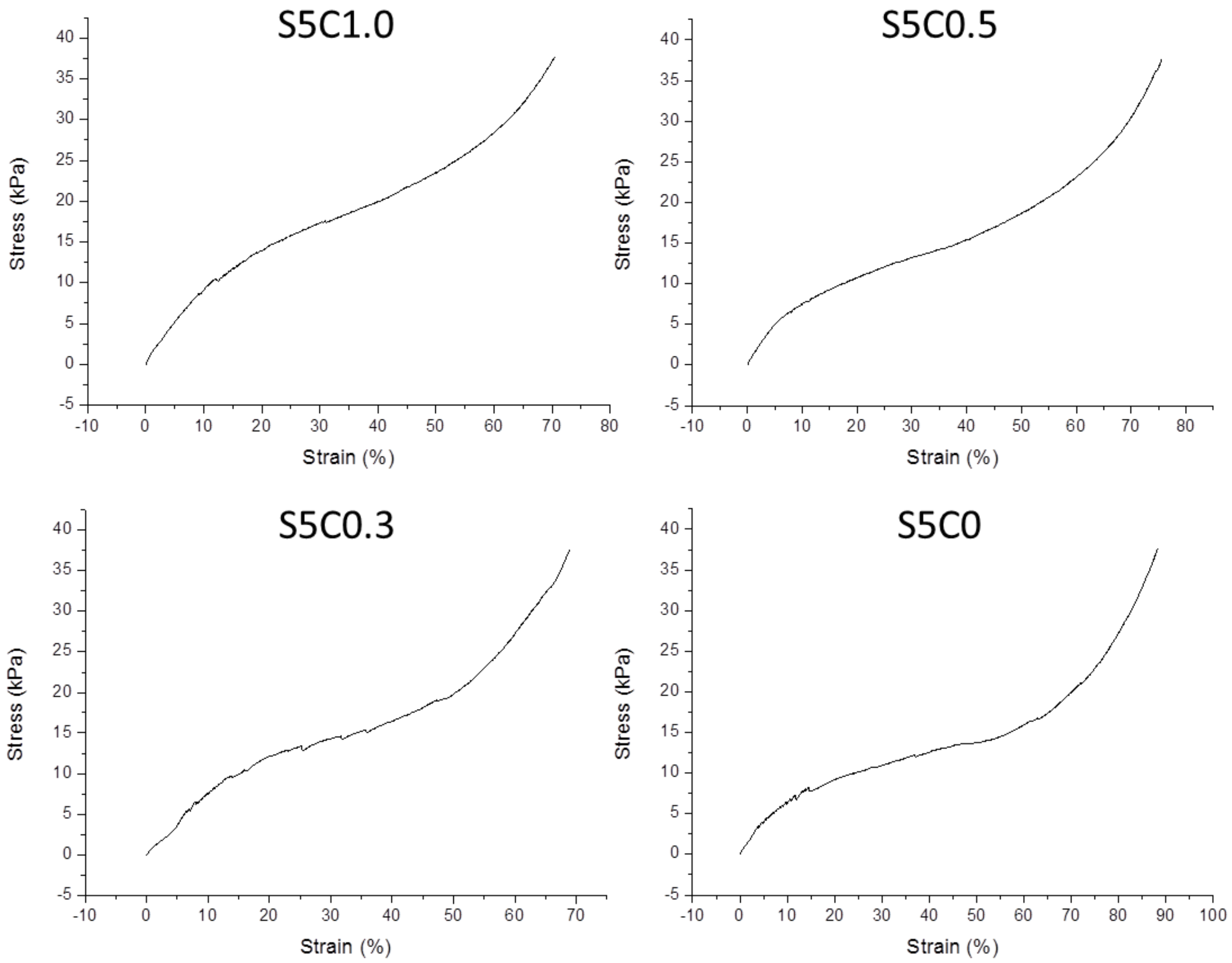

Figure S1: Stress-strain curves of all samples and control. Number of samples measured: S5C1.0 n=5; S5C0.5 n=5; S5C0.3 n=6; S5C0 n=4.

Deconvolution results

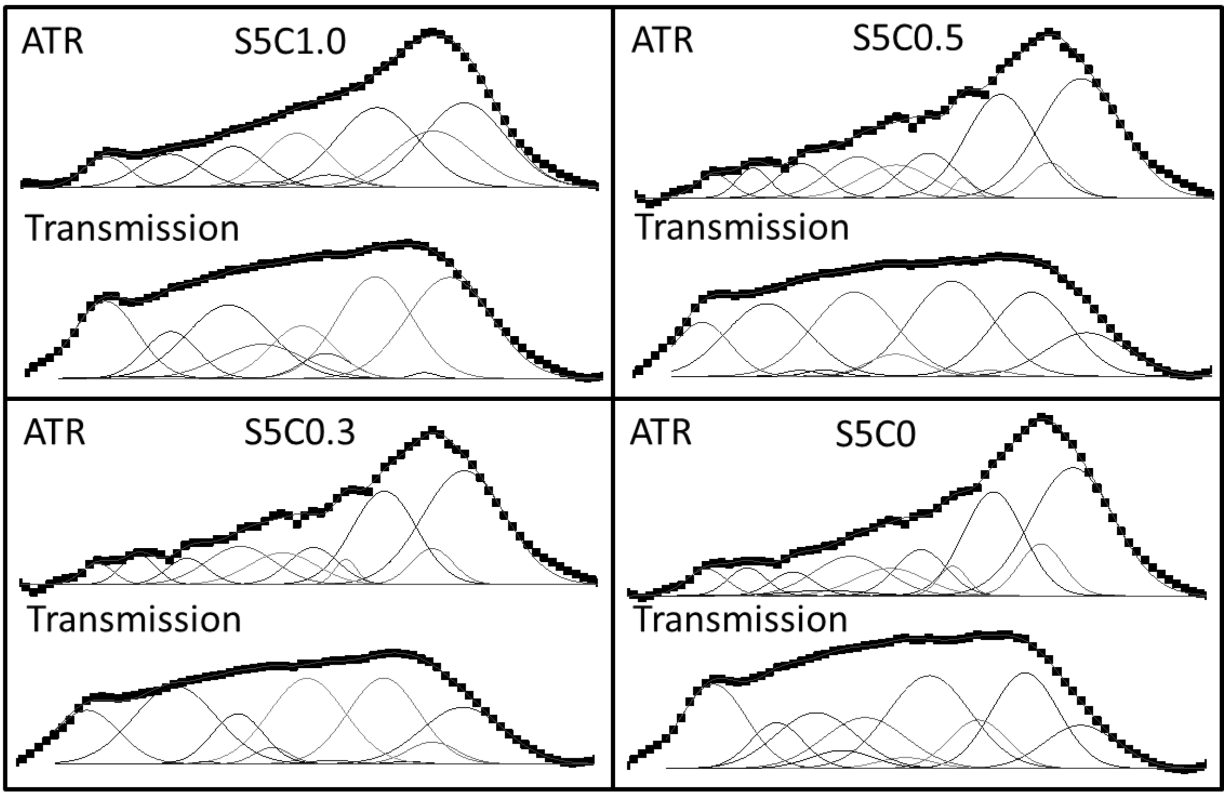

Figure S2: Diagrams of deconvoluted peaks in amide I band region. Bands for peak allocation to secondary structure are: β-sheet: 1611 cm^{-1}, 1619 cm^{-1}, 1626 cm^{-1}, 1699 cm^{-1}. α-helix/random coil: 1631 cm^{-1}, 1641 cm^{-1}, 1649 cm^{-1}. Turns and bends: 1659 cm^{-1}, 1668 cm^{-1}, 1674 cm^{-1}, 1682 cm^{-1}.

SF matrix /cellulose scaffold composite structure

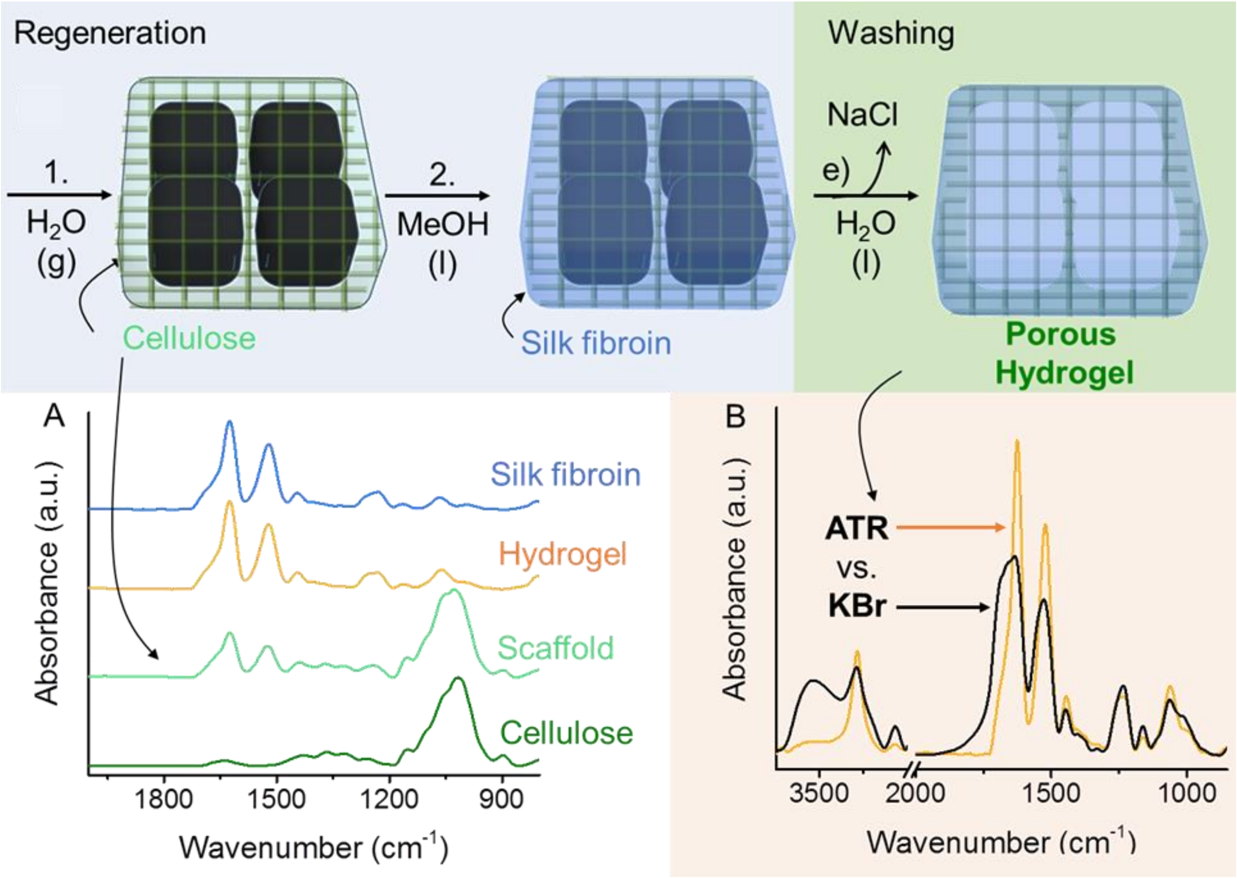

Figure S3: Cellulose framework structure embedded in a SF matrix. (A) Cellulose framework: By omitting methanol treatment of samples, a cellulose-rich, very stable hydrogel formed ("Scaffold" spectrum); that sample was compared with regenerated cellulose, degummed silk fibroin and lyophilized SF/cellulose hydrogel by ATR FTIR. (B) SF matrix: ATR and transmission spectra (KBr) normalized to the cellulose-specific band area 1100 cm^{-1} to 960 cm^{-1} of a lyophilized hydrogel. The larger amide band area in ATR mode, indicates a relatively increased SF content in the surface-near region compared to the bulk.

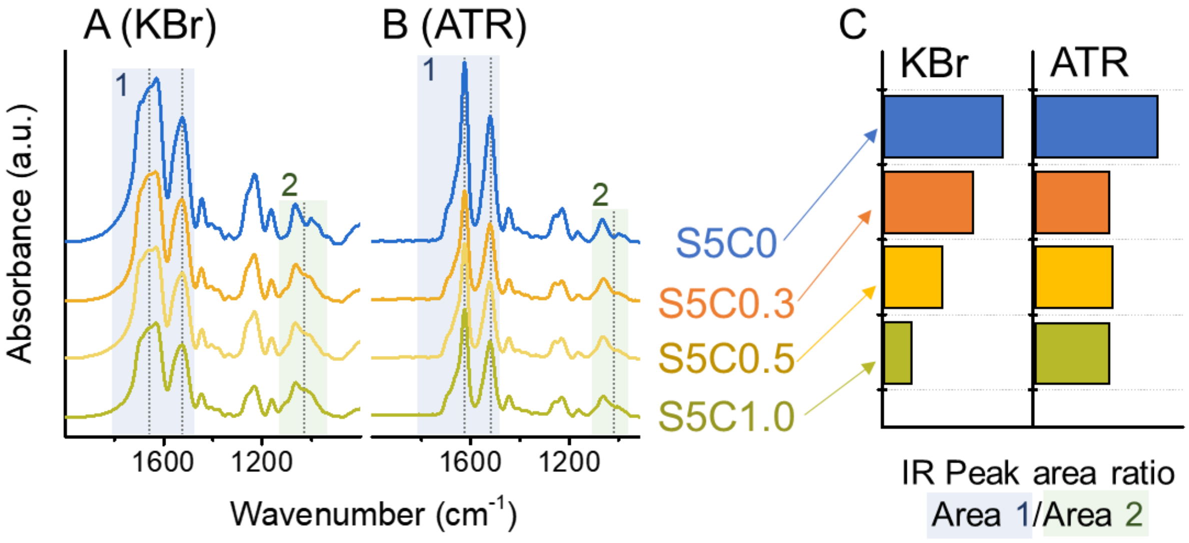

Figure S4: Different bulk and surface composition. Ratios of silk fibroin (SF) specific amide I and II band area and cellulose-specific band area at 1100 cm^{-1} to 960 cm^{-1} (respectively area 1 and 2 in A and B) of all samples decrease with increasing cellulose content in transmission mode (KBr) (C). In ATR mode ratios of samples do not differ, but show a lower ratio compared to the SF control. This indicates a richer SF structure in the surface regions of the hydrogel compared to bulk composition.
